# Comparative analysis of pathogenicity and anticoccidial resistance of field-isolated *Eimeria* strains in Sichuan Province, China

**DOI:** 10.3389/fvets.2025.1692907

**Published:** 2026-01-05

**Authors:** Runhui Zhang, Haitao Li, Xiao Wang, Rihong Jike, Shuaiqian Lu, Lili Hao

**Affiliations:** 1 College of Animal Husbandry and Veterinary Medicine, Southwest Minzu University, Chengdu, China; 2 Agricultural and Rural Bureau of Liangshan Yi Autonomous Prefecture of Sichuan Province, Liangshan, China

**Keywords:** chicken coccidiosis, *Eimeria*, field isolates, drug resistance, anticoccidials

## Abstract

Chicken coccidiosis is an intestinal protozoan disease caused by *Eimeria*, which is distributed worldwide and causes significant economic losses in the poultry industry. Owing to prolonged anticoccidial use, drug resistance has become an important obstacle to the control of coccidiosis. This study aimed to evaluate the pathogenicity of three mixed-species *Eimeria* isolates collected from chicken farms in Sichuan Province, China. The drug resistance of the isolates was also evaluated, including synthetic drugs, ionophores, and a natural herb. A total of 132 newborn chicks were grouped, including an infected-treated group, a positive control for each isolate, and a negative control. Survival rate, weight gain, oocyst excretion, and lesion scores were recorded. The pathogenicity and drug resistance of the three isolates were determined by four indices, including the anticoccidial index (ACI), the optimal percentage of anticoccidial activity (POAA), the relative oocyst production (ROP), and the reduction of lesion score (RLS). The results showed that only one *Eimeria* spp. isolated from Xichang was sensitive to sulfaclozine, whereas the rest of the isolates exhibited resistance to all tested drugs, with most isolates demonstrating severe or complete resistance. These findings are crucial for the selection of a regional drug administration program to control coccidiosis in chickens.

## Introduction

Chicken coccidiosis, caused by various *Eimeria* species, leads to hemorrhagic diarrhea, necrotic enteritis, and growth disorders. Currently, the seven most important *Eimeria* species worldwide are *Eimeria tenella, E. acervulina, E. maxima, E. brunetti, E. mitis, E. necatrix*, and *E. praecox* (1, 2). In recent years, global economic losses attributed to chicken coccidiosis have exceeded USD 10 billion annually (3). In China, coccidia infection rates range from 21.4 to 67.1% (4). Annual economic losses exceed USD 73 million, with treatment costs between USD 73 million and USD 88 million (1, 5). Most poultry operations control coccidiosis using anticoccidials, including polyether ionophores and synthetic compounds. Polyether ionophores are naturally occurring lipid-soluble compounds produced by bacteria that transport cations across biological membranes. Commonly used ionophores include salinomycin (6), monensin (7), and maduramicin (8). Synthetic anticoccidials inhibit the folic acid pathway and thiamine uptake. These include sulfonamides (9), diclazuril (10), and ethanamizuril (11). Furthermore, many natural compounds have been investigated for their anticoccidial properties, including *Artemisia* (12, 13), *Quillaja*, and essential oils of tea trees (13).

Almost all chicken farms rely on anticoccidials as feed additives for chickens, particularly broilers, throughout their lifespan. However, long-term drug use has inevitably led to the emergence of drug-resistant strains, which have severely disrupted the global control of chicken coccidiosis. In 1994, Peeters et al. compared the sensitivity of seven synthetic drugs and five ionophores against isolated coccidia strains in Belgium, indicating a reduced efficacy of both ionophores and synthetic drugs (14). A Colombian survey reported limited diclazuril efficacy against *Eimeria* spp. in 2021 (15). More recently, Flores et al. found that strains isolated from chicken farms in South Korea exhibited severe resistance to six *Eimeria* species, including diclazuril and monensin (16). Drug resistance has impacted the efficacy, safety, and drug residues of anticoccidials in the poultry industry (17). In response, many countries have banned the use of antibiotics as feed additives, such as sulfonamides. European Union (EU) countries have already implemented EU Regulation (EC) No. 1831/2003. Probiotics, prebiotics, and natural herbs are recommended as alternative control strategies (18, 19).

While antibiotics in feed are banned in China, certain anticoccidials remain authorized as feed additives (Regulation No. 194, ‘Ban on the Production of Commercial Feed Containing Antibiotics by Feed Enterprises,’ China). However, data on anticoccidial resistance in Southwest China are lacking. Therefore, we investigated the drug resistance and pathogenicity of three field strains from Sichuan Province. Additionally, a comparative analysis of the pathogenicity of these field isolates was performed.

## Materials and methods

### Animals and ethics

A total of 132 one-day-old Sichuan yellow-feathered hybrid broilers (*Gallus domesticus*, Tianfu Huangji No.3, strain code: TF-3) were purchased from a poultry farm (Xinnong Poultry Farm, Chengdu, China). All chicks were housed in a climate-controlled room with a thermoneutral environment of approximately 30 °C and approximately 65% humidity. A heat lamp was used to maintain a temperature of approximately 40 °C, which was reduced by 2 °C per week in each cage until the chicks reached 15 days of age. The chicks were kept coccidia-free before infection. Unlimited water and food without any anticoccidial additives were supplied for the commercial basic diet (CP Feed, C.P. Pokphand Co. Ltd., Hebei, China). All protocols were approved by the Ethics Committee of Southwest Minzu University in accordance with the recommendation of the Animal Care and Use Program Guidelines of Sichuan Province, China (No. SMU-202401139, September 2024).

### Sample collection, isolation, and propagation of field strains

Fresh fecal samples (50–100 g) were collected from three farms with coccidiosis outbreaks in Xichang (XC), Puge (PG), and Deyang (DY). The samples were brought back to the Laboratory of Parasitology at Southwest Minzu University. Fecal samples were collected from chickens with no history of anticoccidial administration or vaccination. The samples were morphologically identified as *Eimeria* by flotation. Briefly, feces were mixed with sterile distilled water at a ratio of 1:1 and stirred with an electric blender until no large particles were observed. The suspension was centrifuged at 3,000 rpm for 8 min, and the pellet was resuspended in saturated saline. After centrifugation at 3,000 rpm for 8 min, the top layer of the supernatant was checked by microscopy. The samples obtained as *Eimeria*-positive were further isolated, purified, and stored in 2.5% potassium dichromate, as previously described with slight modifications (20). After centrifugation, the saturated saline supernatant containing *Eimeria* was transferred to clean tubes, and nine times the volume of water was added. The oocysts were washed and collected after centrifugation at 3,000 rpm for 8 min. *Eimeria* oocysts were sporulated in 2.5% potassium dichromate solution at 28 °C for 72 h under continuous aeration until reaching a sporulation rate of 95%. The sporulation rate was determined by counting the number of sporulated oocysts per milliliter using a hemocytometer. The oocysts were centrifuged to remove potassium dichromate and inoculated into 14-day-old chicks with no history of coccidiosis to propagate the parasite.

The obtained strains have been identified by PCR previously (21), of which both field isolates of Deyang (DY) and the Pug (PG) contained *E. tenella, E. acervulina*, and *E. maxima*. The field strains of Xichang (XC) included *E. tenella* and *E. acervulina*.

### Anticoccidials

Six anticoccidials were used to evaluate the drug resistance of the field strain. Briefly, the powder extract of *Artemisia annua L* was purchased from Shandong Jieren Bioengineering Co., Ltd. (Shandong, China); administration: 2 g/kg mixed in feed. Sulfaclozine powder was purchased from Hefei Xinkexin Animal Pharmaceutical Co., Ltd. (Hefei, China), administration: 2 g/kg mixed in feed. Salinomycin was purchased from Dalian Xinwei Biotechnology Co., Ltd. (Dalian, China); administration: 60 mg/kg mixed in feed. Diclazuril was purchased from Sichuan Tongda Animal Health Technology Co., Ltd. (Sichuan, China); administration: 1 mg/kg mixed in feed. Ethanamizuril was purchased from Zhongmu Anda Pharmaceutical Co., Ltd. (Hubei, China); administration: 1 mL/L mixed in drinking water. Amprolium hydrochloride was purchased from Henan Huikang Animal Pharmaceutical Co., Ltd. (Henan, China); administration: 0.5 mg/L mixed in drinking water.

### Experimental design

At 12 days of age, the birds (broilers, *n* = 132) were confirmed to be *Eimeria*-free before the trials. All experimental birds were stratified by body weight and randomly assigned to 10 groups to ensure that the average body weight of each group was essentially consistent. Specifically, six groups were treated with each anticoccidial agent (infected-treated groups, *n* = 18 per group), three positive controls were infected with each *Eimeria* isolate (DY, PG, and XC) without treatment (PCs, *n* = 6 per group), and one blank control (NC, *n* = 6). Birds in each group were randomly housed in separate cages labeled with only group codes to minimize human bias. Throughout the trial, the birds in the infected-treated group received the recommended dosage of anticoccidials according to the manufacturer’s instructions.

At 14 days of age, each infected-treated group was further divided into three subgroups (DY, PG, and XC), with six birds in each replicate. Then, each bird was orally inoculated with 8 × 10^4^ sporulated oocysts. Clinical observations were recorded daily throughout the trial period, including bloody diarrhea, abnormal conditions, growth, and mortality. Feces were collected daily from 4 to 8 days post-infection (dpi) to determine the number of oocysts per gram of feces (OPG) using McMaster’s counting method. Briefly, 2 g of fresh feces was homogenized with 10 mL of saturated saline in a mortar, followed by adding 50 mL of saturated saline. The mixture was filtered through a sieve, and the resulting suspension was thoroughly mixed before being transferred to the chambers of a McMaster counting slide. Oocysts were counted twice at 10× magnification under a microscope after 5 min of sedimentation at room temperature. Dilution was performed if the oocyst numbers were too high for accounting. OPG = [(oocyst number in chamber 1 + oocyst number in chamber 2)/(2 × 0.15)] × 10.

All birds were weighed again and then gently euthanized at 8 dpi by being placed in a closed chamber where the concentration of carbon dioxide was gradually increased over 5 min. Then, coccidia lesions in the entire intestinal tract were examined and averaged, as previously described (22). Specifically, the cecum was examined for lesions caused by *E. tenella* infection, the duodenum and anterior small intestine were examined for *E. acervulina* infection, and the mid-small intestine was examined for *E. maxima* infection. Lesions in infected birds that died were scored as 4.

### Evaluation of anticoccidial resistance

Four indices were used to evaluate drug resistance (23): the percent of optimum anticoccidial activity (POAA), the reduction of lesion scores (RLS), the relative oocyst production (ROP), and the anticoccidial index (ACI).

POAA = (GSR of infected-treated group - GSR of PC group) ÷ (GSR of NC group - GSR of PC group) × 100%. Growth and survival ratio (GSR) = final weight/initial weight. POAA >50% was considered sensitive, and POAA ≤ 50% was considered resistant (24).

RLS = (mean of PC group - mean lesion score of infected-treated group)/mean lesion score of PC group × 100. RLS > 50% was considered sensitive, and RLS ≤ 50% was considered resistant (25).

ROP = (average oocyst output of the infected-treated group/average oocyst output of the PC group) × 100%. ROP ≥ 15% was considered resistant, and ROP < 15% was sensitive (26).

ACI = (survival rate + relative body weight gain) - (lesion value + oocyst value). Survival rate = (number of surviving chickens/total number of chickens) × 100. Relative weight gain rate = (average weight gain of the infected group/average weight gain of the NC group) × 100. Lesion value = lesion score × 10. The oocyst value was determined based on the percentage of ROP. Specifically, 0–1.0% corresponds to value 0, 1.0–25.0% corresponds to value 5, 26.0–50.0% corresponds to value 10, 51.0–75.0% corresponds to value 20, and 76.0–100% corresponds to value 40. ACI ≥ 160 was considered sensitive, whereas ACI < 160 was considered resistant to anticoccidials (22).

The final evaluation of drug resistance was determined as follows: no drug resistance when all four indices were considered sensitive (−), mild resistance (+) when only one index was considered resistant, moderate resistance (++) when two indices were considered resistant, severe resistance (+++) when three indices were considered resistant, and complete resistance (++++) when all four indices were considered resistant.

### Statistical analysis

Statistical analyses were performed using GraphPad Prism 5. Group differences were compared using Student’s *t*-test, and differences among drugs were compared using Tukey’s multiple comparison test. A *p* value of <0.05 was considered statistically significant.

## Results

### Comparison of the pathogenicity of three *Eimeria* spp. isolates

The following section describes the pathogenicity and resistance of the three field isolates. During the trial, all infected birds showed typical clinical signs, such as decreased intake of water and food, lethargy, and hemorrhagic diarrhea. In terms of weight gain, the PG isolate showed the highest mean weight gain at 150.6 g, whereas the XC isolate group had the lowest mean weight gain at 110.0 g (Table 1). The lesion score of DY isolates was the highest (2.7 ± 0.8). However, no statistically significant differences were observed among the three isolates (*p* > 0.05). The highest mean OPG was recorded in the PC_XC group, accounting for 28.3 ± 23.8 × 10^4^. In addition, two birds in the PC_XC group died during the study period (Table 2).

**Table 1:** Relative weight gain, lesion score, and OPG of each *Eimeria* isolate.

Groups	DY			PG			XC		
	Mean body weight gain (g)	Lesion score (±SD)	Mean OPG ×10^4^ (±SD)	Mean body weight gain (g)	Lesion score (±SD)	Mean OPG ×10^4^ (±SD)	Mean body weight gain (g)	Lesion score (±SD)	Mean OPG ×10^4^ (±SD)
PC	111.4	2.7 ± 0.8	25.3 ± 20.9	150.6	2.0 ± 0.3	20.1 ± 11.3	110.0	2.3 ± 1.3	28.3 ± 23.8
Sulfaclozine	123.0	1.2 ± 0.4^**^	5.5 ± 2.6	118.1	1.8 ± 0.5	1.6 ± 1.6^**^	211.2	1.2 ± 1.5	0.2 ± 0.2^**^
Diclazuril	145.7	2.2 ± 1.2	20.8 ± 13.7	84.6	1.5 ± 0.7	20.5 ± 14.5	84.7	2.3 ± 1.4	16.4 ± 11.1
*Artemisia annua*	123.0	1.7 ± 0.5	5.0 ± 1.1	144.6	0.5 ± 0.4^*^	12.7 ± 8.4	103.1	1.9 ± 1.6	6.0 ± 2.2
Ethanamizuril	142.0	2.0 ± 0.9	13.0 ± 8.6	171.6	1.9 ± 0.6	15.6 ± 12.5	119.1	2.4 ± 1.3	3.0 ± 1.4^*^
Salinomycin	190.8	1.3 ± 0.8^*^	9.3 ± 6.1	157.6	1.8 ± 0.6	3.9 ± 1.2^*^	218.5	1.1 ± 0.2	10.1 ± 5.5
Amprolium hydrochloride	200.0	1.0 ± 0.0^**^	32.4 ± 19.0	120.8	1.1 ± 0.4^**^	12.1 ± 5.0	106.5	0.6 ± 0.4^*^	24.7 ± 22.5

^*^p < 0.05, ^**^p < 0.01 compared to the PC group of each isolate.

**Table 2:** Relative weight gain rate, survival rate, lesion value, and oocyst value of anticoccidials.

Groups	DY				PG				XC			
	Relative weight gain rate (%)	Survival rate (%)	Lesion value	Oocyst value	Relative weight gain rate (%)	Survival rate (%)	Lesion value	Oocyst value	Relative weight gain rate (%)	Survival rate (%)	Lesion value	Oocyst value
Sulfaclozine	57.6	100.0	16.7	5	54.6	100.0	17.5	5	94.6	83.3	11.7	5
Diclazuril	37.6	100.0	21.7	40	65.3	100.0	15.0	40	47.9	66.7	23.3	20
*Artemisia annua*	64.5	100.0	16.7	5	58.7	100.0	5.0	20	46.2	66.7	19.2	5
Ethanamizuril	76.9	83.3	20.0	20	63.8	100.0	19.2	40	53.3	66.7	23.3	5
Salinomycin	90.9	100.0	13.3	10	85.8	100.0	18.3	5	97.9	100.0	10.8	10
Amprolium hydrochloride	54.1	100.0	10.0	40	89.4	100.0	10.8	20	77.7	100.0	5.8	40
PC	50.0	100.0	27.1	40	61.5	100.0	20.0	40	49.3	66.7	23.3	40

### The anticoccidial index (ACI)

The anticoccidial index (ACI) was calculated using the relative weight gain, survival rate, lesion value, and oocyst value based on raw data, including mean body weight gain, lesion scores, and oocyst output (Table 1). Regarding mean body weight gain, birds treated with amprolium hydrochloride showed the highest weight gain, which was nearly twice that of the PC group after infection with the DY isolate. Birds infected with the PG isolate showed the lowest weight gain after diclazuril treatment. For the XC isolate, salinomycin-treated birds exhibited the highest weight gain at 211.2 g. For lesion scores, significant reductions were observed in birds treated with sulfaclozine (*p* < 0.01, *t*(30) = 2.872), salinomycin (*p* < 0.05, *t*(10) = 2.880), and amprolium hydrochloride (*p* < 0.01, *t*(10) = 4.986) in the DY group. In the PG group, lesion scores decreased significantly in birds treated with *A. annua* (*p* < 0.05, *t*(10) = 2.880) and amprolium hydrochloride (*p* < 0.05, *t*(10) = 4.536). Only the amprolium hydrochloride-treated birds showed a significant reduction (*p* < 0.05, *t*(10) = 3.099) in lesion scores compared to the PC group for the XC isolates. For oocyst output, no significant differences were observed among the groups infected with the DY isolate (*p* > 0.05). However, birds treated with sulfaclozine (*p* < 0.01, *t*(8) = 3.626) and ethanamizuril (*p* < 0.05, *t*(8) = 0.591) shed a significantly reduced number of oocysts in the PG group. The oocyst output decreased significantly in the XC group treated with sulfaclozine (*p* < 0.01, *t*(8) = 2.664) and ethanamizuril (*p* < 0.05, *t*(8) = 2.379). Survival data showed no mortality in the PG group, with only one bird dying in the PC group following administration (Table 2). In the PG group, 100% survival was exclusively observed in birds treated with salinomycin and amprolium hydrochloride.

**Figure 1:**
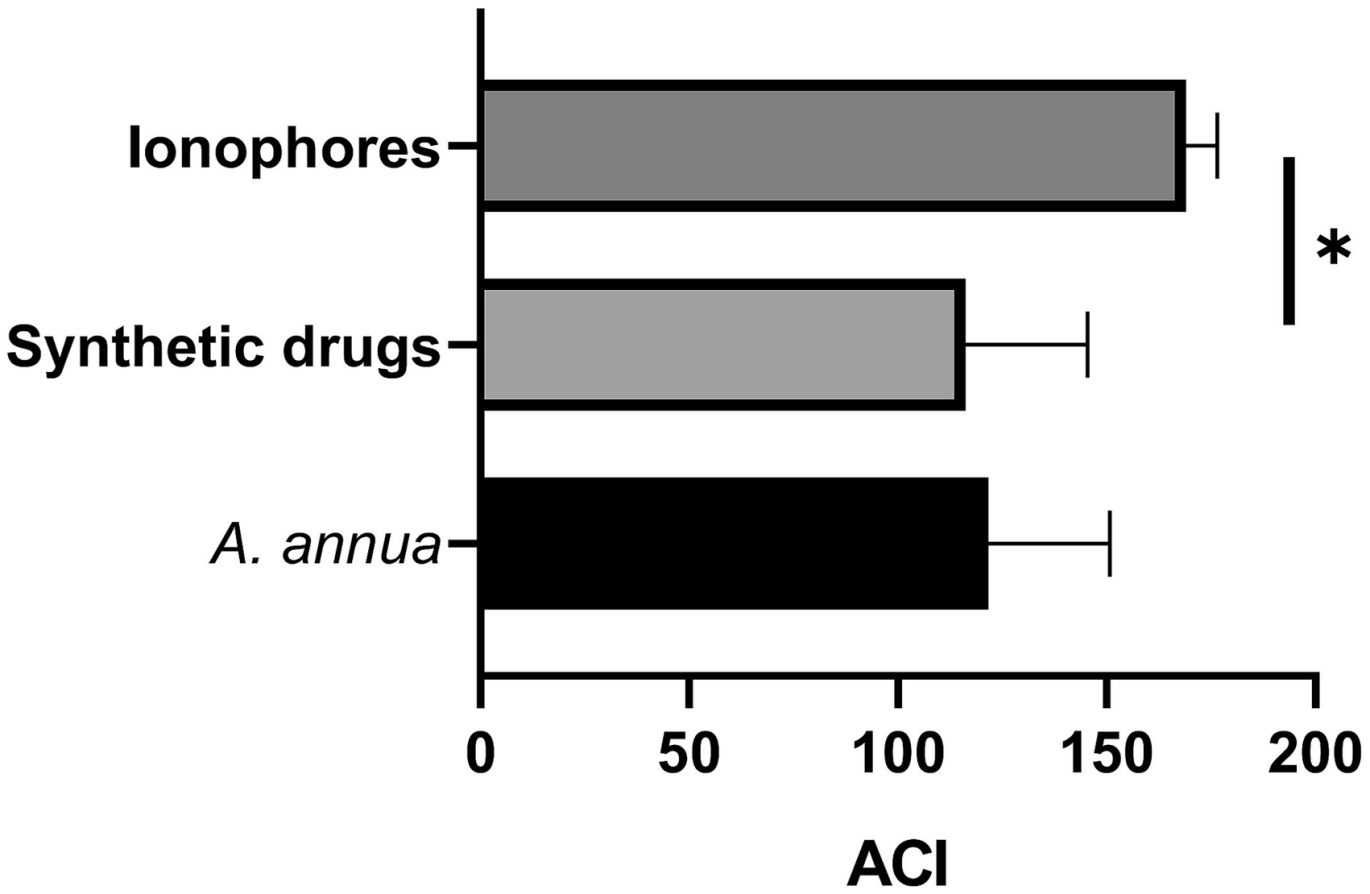
Comparison of drug efficacy among three agents (*A. annua*, synthetic drugs, and ionophores) based on ACI values. *p < 0.05 and **p < 0.01 refer to the results of pairwise comparisons between drugs.

The ACI was determined to be 200 for the NC group. Based on the anticoccidial index (Table 3, Figure 1), three *Eimeria* spp. isolates were resistant to most of the tested anticoccidials (ACI < 160). The DY and PG isolates showed sensitivity to salinomycin, with ACI values of 167.60 and 162.50, respectively. The XC isolate was sensitive to sulfaclozine (ACI = 169.60) and salinomycin (ACI = 177.09).

**Table 3:** Anticoccidial index (ACI), percentage of optimum anticoccidial activity (POAA), reduction in lesion scores (RLS), and relative oocyst production (ROP) for each isolate.

Groups	DY					PG					XC				
	ACI	RLS (%)	ROP (%)	POAA (%)	Total drug resistance	ACI	RLS (%)	ROP (%)	POAA (%)	Total drug resistance	ACI	RLS (%)	ROP (%)	POAA (%)	Total drug resistance
Sulfaclozine	135.9	**55.6**	21.7	1.9	severe (+++)	132.1	11.1	**7.9**	−20.9	severe (+++)	**161.3**	**50.3**	**0.7**	**65.9**	sensitive (−)
Diclazuril	75.9	18.5	82.3	−26.1	complete (++++)	110.3	23.0	102.1	11.1	complete (++++)	71.3	0.0	57.8	−3.0	complete (++++)
*Artemisia annua*	142.8	37.0	19.6	18.4	complete (++++)	133.7	**74.6**	63.0	−8.2	severe (+++)	88.7	−19.8	21.0	−8.8	complete (++++)
Ethanamizuril	120.2	25.9	51.4	**52.7**	severe (+++)	104.6	7.1	77.8	−0.3	complete (++++)	91.7	0.0	**10.5**	3.5	severe (+++)
Salinomycin	**167.6**	**51.9**	37.0	26.3	moderate (++)	**162.5**	11.1	19.6	**56.2**	moderate (++)	**177.1**	**53.2**	35.5	**88.4**	mild (+)
Amprolium hydrochloride	101.5	**63.0**	128.2	−33.3	severe (+++)	158.6	44.4	60.0	**74.9**	severe (+++)	131.9	**73.2**	87.0	47.3	severe (+++)
PC	82.9	0.0	100.0	0.0	−	107.5	0.0	100.0	0.0	−	52.6	0.0	100.0	0.0	−

The number with a bold represents negative, (−) represents sensitive, (+) represents mild resistance, (++) represents moderate resistance, (+++) represents severe resistance, and (++++) represents complete resistance. ACI≥160 was considered sensitive, whereas ACI <160 was considered resistant to anticoccidials; POAA >50% was considered sensitive, whereas POAA ≤50% was considered resistant. RLS >50% was considered sensitive, whereas RLS ≤50% was considered resistant; ROP≥15% was considered resistant, whereas ROP <15% was considered sensitive. DY, *Eimeria* isolate from a chicken farm in Deyang; XC, *Eimeria* isolate from a chicken farm in Xichang; PG, *Eimeria* isolate from a chicken farm in Puge; ACI, the anticoccidial index; POAA, the optimal percentage of anti-coccidiosis activity; ROP, the relative oocyst production (ROP); RLS, the reduction of lesion score.

### Percent of the optimum anticoccidial activity (POAA), the reduction of lesion scores (RLS), and the relative oocyst production (ROP)

The POAA value was calculated based on body weight gain data. The DY isolate was sensitive to ethanamizuril (Table 3). The PG isolate was sensitive to salinomycin and amprolium hydrochloride. The XC isolate was sensitive to sulfaclozine and salinomycin but resistant to the remaining drugs. Three of the isolates showed resistance to diclazuril and *A. annua*. The ROP value was calculated based on the oocyst output data. Specifically, the DY isolate was resistant to all six drugs (Table 3). The PG isolate was sensitive to sulfaclozine, whereas the XC isolate was sensitive to sulfaclozine and ethanamizuril, according to ROP values. RLS value was calculated based on lesion scores. The PG isolate was identified as resistant to all anticoccidials except *A. annua* based on the RLS value. The DY and XC isolates showed resistance to diclazuril, *A. annua*, and ethanamizuri.

### Evaluation of the anticoccidial resistance

After comprehensive evaluation using the four indices (Table 3), all isolates were resistant to the tested drugs, except the XC isolate, which was sensitive to sulfaclozine. Regarding the resistance of three field isolates to the six drugs, DY and PG isolates were severely resistant to sulfaclozine. All three isolates were completely resistant to diclazuril. Resistance to *Artemisia* varied among all isolates, ranging from severe to complete. DY and XC isolates were severely resistant, and PG was completely resistant to ethanamizuril. XC isolate was mildly resistant, while DY and PG were moderately resistant to salinomycin. All isolates were severely resistant to amprolium hydrochloride. In a comparison of efficacy among *A. annua*, synthetic drugs, and ionophores, ionophores showed significantly higher ACI values compared to synthetic drugs (*p* < 0.05). *A. annua* exhibited lower efficacy than ionophores, but with no significant difference.

## Discussion

This study demonstrated high multidrug resistance among three field-isolated *Eimeria* strains from the Sichuan Province, China. Chicken coccidiosis is prevalent in both intensive and free-range chicken farms in China. In this study, we used cage feeding and evaluated four standard indices (i.e., ACI, POAA, ROP, and RLS) to assess the pathogenicity of three *Eimeria* isolates and the drug resistance of six anticoccidials, including ionophores, synthetic drugs, and a natural herb. Based on the pathogenicity results, the XC isolate displayed the highest pathogenicity and the lowest survival rate. However, no significant differences were observed in oocyst shedding or in intestinal lesions. The XC isolates contained only two *Eimeria* species, whereas DY and PG each contained three species. Using mixed *Eimeria* species during the trial, particularly the presence of species with greater reproductive potential, could influence the final outcome (27). We hypothesized that, with the same total infection dose of mixed strains, the infection load per species in the XC strain was relatively higher, leading to increased pathogenicity. Additionally, *E. acervulina*-induced lesions did not reach a peak lesion score at 8 dpi. Therefore, the assessment of intestinal lesions is less precise compared to using single-species isolates. Although isolating individual species and further evaluating drug efficacy for each species may yield more precise results, we chose mixed *Eimeria* isolates for testing drug resistance, primarily to simulate the mixed infection that commonly occurs in poultry farms.

The control and treatment of chicken coccidiosis have relied on the use of anticoccidials for a long time. The challenge of drug resistance has existed in China since 1994 (28). Polyether ionophores and synthetic compounds are the major categories of anticoccidials commonly used in China (29). Additionally, some natural compounds have also been applied for the treatment and control of chicken coccidiosis in China, such as artemisinin (30) and halofuginone (31). All the *Eimeria* isolates displayed resistance to six anticoccidial drugs, except for the XC isolate, which was sensitive to sulfaclozine. Most isolates showed moderate to complete resistance to the tested anticoccidials. Furthermore, multidrug resistance was present in all field isolates, which is consistent with previous studies (16, 32). A recent study indicated that isolated *Eimeria* spp. exhibited medium to complete resistance to 11 anticoccidial drugs from six chicken farms located in central Sichuan, China (33), which was similar to our findings.

Sulfonamides were the first effective drugs used to control chicken coccidiosis, and the development of resistance has been investigated for decades. A previous study reported that sulfonamides attributed activity against *Eimeria* species such as *E. acervulina, E. maxima*, and *E. brunetti*, but not against *E. tenella* or *E. necatrix* (34). However, the XC isolate, containing *E. tenella* and *E. acervulina*, revealed sensitivity to sulfaclozine. Antibiotics have been prohibited as feed additives in China since 2020. Sulfonamides remain permitted for treating chicken coccidiosis and bacterial infections in livestock; however, they are not recommended. Prior to 2020, sulfonamides were among the most frequently detected antibiotics in the manure of chickens, cattle, and pigs in China (35). In this study, only one of the three isolates showed sensitivity to sulfaclozine, possibly due to policy implications. Consequently, two sulfonamide-resistant isolates in the current study indicated that the issue of antibiotic misuse may still persist, possibly impacting animal health due to ineffective treatment, increasing environmental pollution, and reducing meat production (36).

Ethanamizuril, a novel triazine anticoccidial compound developed by the Shanghai Veterinary Research Institute of the Chinese Academy of Agricultural Sciences (37), has recently been commercially produced and used in the poultry industry in China. It has been found to effectively inhibit the development of secondary generation merozoites and early stages of gametogenesis in *E. tenella* (38). An *in vivo* study of anticoccidial sensitivity demonstrated that it is not only safe but also highly effective, with an ACI > 180 (39). However, little is known about the current resistance of the prevalent strains in China. Interestingly, all three isolates were more than severely resistant, despite none of the farms having a history of exposure to ethanamizuril in this study. Previous studies have indicated that *Eimeria* isolates resistant to one ionophore are cross-resistant to other compounds with similar modes of action (32, 40). Diclazuril and toltrazuril, both triazine anticoccidial compounds, are widely used in the poultry industry in China. In this study, we evaluated the resistance of each isolate to diclazuril. The results demonstrated that all three isolates were completely resistant to diclazuril. If the *Eimeria* strain develops resistance to multiple drugs simultaneously, this may lead farmers to use higher doses, thereby increasing the risk of drug residues and posing a threat to food safety. It is recommended to rotate drugs regularly to avoid the use of similar compounds within a short period. These findings suggest that further studies on triazine compounds could enhance our understanding of the mechanisms underlying drug resistance.

In recent years, plant extracts and essential oils have been introduced as alternatives for the control of chicken coccidiosis. Specifically, *Artemisia* has been shown to reduce the number of *Eimeria* oocysts both *in vitro* and *in vivo* (13, 41). A recent field trial indicated that *A. annua* showed promising results in the control of coccidiosis, including the reduction of oocyst output and lesion score and improved feed conversion in broilers (42). Currently, little is known about drug resistance associated with *Artemisia*, despite its use in the field for several years. A recent study on drug resistance showed that the *E. tenella* field strain displayed moderate resistance to *Artemisia* from a chicken farm in Xuzhou (43). In this study, we observed reduced OPG with no significant difference among the three isolates, while the PG isolate showed a significant reduction in lesion scores compared to the PC group. Based on the assessment of the four indices, the XC isolate showed severe resistance, and the PG and DY isolates were completely resistant. Thus, these data indicated that natural herbs are not deemed to induce resistance as an alternative; however, the emergence of resistance cannot be entirely precluded. However, their efficacy was previously found to be lower than that of antibiotics (44). Combining ionophores with natural herbs may yield better outcomes. For example, combining *Artemisia* and monensin has been shown to improve growth performance and significantly reduce OPG in broilers (45).

This study employed standardized methods for the evaluation of anticoccidial resistance, including relative weight gain, survival rate, lesion score, and oocyst output, which are parameters widely used in drug efficacy assessments. This study provides an initial insight into drug resistance. To inform practical administration strategies, it is advisable to expand the sample size, such as by conducting an evaluation of drug efficacy in specific chicken houses on farms. The emergence of drug resistance is primarily because of the increased and unregulated use of potent anticoccidials (46). The development of drug resistance can be very fast, such as with quinolones and pyridinols (47). Drug-resistant strains readily disseminate because of the rapid reproduction of *Eimeria*. Drug combination is an effective strategy to reduce or postpone the development of resistance. Specifically, combinations of nicarbazin and monensin have been considered effective in controlling ionophore-resistant strains (48). Furthermore, rotation programs incorporating multiple drugs and vaccines have been widely employed to reduce drug sensitivity in chicken farms (49). In this study, ionophores showed better efficacy in controlling coccidiosis than synthetic drugs. Therefore, an ionophore-herbal combination with a rotation program is recommended for this region. Most importantly, the establishment of a regular monitoring protocol is necessary, which should include the assessment of oocyst output, intestinal lesion scores, weight gain, and other key parameters. These findings underscore the urgent need to revise anticoccidial programs in Sichuan Province to reduce resistance development.

## Conclusion

In conclusion, these findings indicated that all *Eimeria* isolates were multidrug resistant to anticoccidials. These insights could help improve drug administration programs to control coccidiosis in chickens in this region. Further research into the drug resistance of individual *Eimeria* species will facilitate a deeper comprehension of species-specific differences in this regard.

## Data Availability

The original contributions presented in the study are included in the article/supplementary material, further inquiries can be directed to the corresponding author.
